# Innovative Use of Fostamatinib in Managing Refractory Immune Thrombocytopenia in an HIV‐Positive Patient: A Case Report

**DOI:** 10.1002/ccr3.70577

**Published:** 2025-06-26

**Authors:** Matteo Bellia, Riccardo Moia, Mattia Schipani, Carola Sella, Mariangela Greco, Wael Al Essa, Abdurraouf Mokhtar Mahmoud, Gianluca Gaidano, Andrea Patriarca

**Affiliations:** ^1^ Division of Hematology, Department of Translational Medicine Universita' del Piemonte Orientale and Azienda Ospedaliero‐Universitaria Maggiore Della Carità Novara Italy

**Keywords:** ART, fostamatinib, HIV infection, immune thrombocytopenia

## Abstract

Managing immune thrombocytopenia (ITP) in HIV patients remains challenging due to refractory cases and treatment limitations. This report highlights fostamatinib as a safe, effective, and antiretroviral therapy‐compatible option, offering durable platelet stabilization in a difficult‐to‐treat population.

**Abbreviations**
ACA: anti‐cardiolipin antibody
ANA: anti‐nucleus antibody
anti‐SSB: anti‐Sjogren's syndrome type B
ART: antiretroviral therapy
C3: complement factor 3
C4: complement factor 4
CT: computed tomography
CYP3A4: cytochrome P450 3A4 isoform
ENA: extractable nuclear antigen
Hb: hemoglobin
HEENT: Head, ears, eyes, nose, throat
HIV: human immunodeficiency virus
HIV‐TP: HIV‐related thrombocytopenia
ITP: immune thrombocytopenia
IVIG: intravenous immunoglobulin
LAC: lupus anticoagulant antibody
Scl‐70: anti‐topoisomerase I antibody
Syk‐I: Spleen Tyrosin Kinase Inhibitor
TPO‐RA: thrombopoietin receptor agonists
WBC: white blood cell

## 1 Introduction

Immune thrombocytopenia (ITP) is a common haematologic complication in patients affected by human immunodeficiency virus (HIV) infection [1, 2]. It can present at any stage of HIV infection and significantly impacts management and prognosis. The pathophysiology of thrombocytopenia in HIV is multifactorial: first, HIV can directly infect megakaryocytes, leading to impaired platelet production and bone marrow suppression [3]; second, the immune response to HIV may result in the production of antiplatelet antibodies, causing increased platelet destruction [2]. Moreover, cytokine dysregulation and chronic immune activation also play crucial roles in the development of thrombocytopenia in HIV‐infected individuals. Thrombocytopenia in HIV patients was first recognized in the early years of the HIV epidemic, and, despite advancements in antiretroviral therapy (ART), its prevalence remains of clinical relevance [4, 5]. It is also associated with increased morbidity, including an increased risk of bleeding and the potential for more severe complications such as thrombotic microangiopathy [6, 7].

The management of thrombocytopenia in HIV patients should address both the underlying HIV infection and the specific haematologic abnormality. Initiation or optimization of ART is critical, as it can lead to significant improvements in platelet counts [4, 6, 8]. In cases where thrombocytopenia persists or is severe, adjunctive treatments such as corticosteroids and intravenous immunoglobulins (IVIG) may be considered [9]. However, patients failing ITP first‐line therapy represent an unmet clinical need, and new therapeutic strategies compatible with ART are necessary and crucial: thrombopoietin receptor agonists (TPO‐RAs) may be useful [10, 11], but the indication of some TPO‐RAs in clinical practice is often strictly limited because of interaction with ART. In this specific setting, fostamatinib, a first‐in‐class Spleen Tyrosin Kinase Inhibitor (Syk‐I), might represent a valid and promising therapeutic option [12, 13, 14, 15].

This case study presents the innovative use, the safety and therapeutic potential of fostamatinib in relapsed and refractory ITP in a 50‐year‐old HIV positive male patient.

## 2 Case Presentation

A 50‐year‐old Caucasian male was referred to our Hematology Clinic in June 2022 because of the incidental detection of isolated thrombocytopenia (platelets count 9.000/μL) in blood tests. He denied clinical signs or symptoms compatible with haemorrhagic diathesis, such as spontaneous epistaxis, gingivorrhagia, melena, petechiae, and ecchymosis. In May 2022, the patient was newly diagnosed with HIV infection. HIV western blot showed marked positivity for P24, P31, P51, GP41, SGP120, weak positivity for SGP105, and negativity for P17, GP36. HIV‐RNA was equal to 80.113 copies/mL and CD3+/CD4+ lymphocyte count was 119/μL (14.8% of all lymphocyte count). Therefore, an antiretroviral therapy (ART) based on bictegravir 50 mg—emtricitabine 200 mg—tenofovir alafenamide 25 mg daily was started with adequate infection control. In June 2022, the HIV‐RNA was decreased to 26 copies/mL and CD3+/CD4+ lymphocyte count was increased to 311/μL.

He referred previous surgical interventions during childhood such appendectomy and tonsillectomy without hemorrhages of clinical note. He denied any pre‐existent autoimmune diseases. Moreover, he reported no allergies to drugs or environmental allergens. In addition, he denied any previous haematologic disorder. Regarding his social habits, he confirmed normal physical activity, a balanced alimentation, no alcoholic abuse nor smoking cigarettes. Given the severe thrombocytopenia, the patient was admitted in the Hematology ward.

On admission, the patient's initial vitals were unremarkable, with a blood pressure of 120/70 mmHg, heart rate of 80′, respiration rate of 13′, temperature of 37.0°C and an oxygen saturation of 98% on ambient air. Physical examination did not reveal muco‐cutaneous haemorrhagic diathesis. No lymphadenopathies were palpable superficially. Head, ears, eyes, nose, throat (HEENT), neck, cardiovascular, respiratory, abdominal, musculoskeletal, and neurological examinations were all grossly unremarkable.

Initial blood test indicated an isolated thrombocytopenia with platelets count 9.000/μL, while white blood cell (WBC) count, hemoglobin (Hb) level, lactate dehydrogenase, vitamin B12, folic acid and coagulation tests were all within the normal range. To better clarify the etiology of this finding, given the HIV positivity, the patient was tested for various infections, including hepatitis C virus, hepatitis B virus, cytomegalovirus, Epstein–Barr virus and *Helicobacter pylori* all returning negative. Furthermore, an autoimmune screening panel was performed: complement factor 3 (C3), complement factor 4 (C4), anti‐nucleus (ANA), anti‐Sjogren's syndrome type B (anti‐SSB), anti‐topoisomerase I (Scl‐70), anti‐cardiolipin (ACA), anti‐beta‐2‐glicoprotein and lupus anticoagulant (LAC) antibodies resulted negative.

These findings suggested a clinical case of severe ITP during HIV infection in active treatment with ART.

## 3 Disease Management and Outcome

Given the severity of thrombocytopenia, the patient underwent a first‐line treatment with high dose methylprednisolone 1 mg/kg intravenously daily and immunoglobulins 1 g/kg intravenously for 2 days. He achieved an initial complete response in terms of platelet count which raised rapidly, so he was discharged after few days in good clinical conditions, continuing an equivalent dose of oral prednisone therapy as outpatient. However, a month later, in July 2022, a new worsening of the platelet count occurred (platelets count equal to 27.000/μL), thus a second line treatment was necessary. A bone marrow evaluation and a total body computed tomography (CT) scan with contrast medium were performed and an underlying lymphoproliferative disorder and/or a secondary cancer were excluded. Specifically, the bone marrow evaluation showed an increased number of megakaryocytes with no evidence of dysplasia, all elements coherent with the diagnosis of ITP. Therefore, considering the concomitant ART and its possible drug interactions, in agreement with the infectious disease consultant, a second‐line treatment based on the TPO‐receptor agonist (TPO‐RA) romiplostim 1 μg/kg subcutaneously once a week was started. The patient achieved a complete response and platelet counts have remained stable in normal range for approximately 6 months. In March 2023, the platelet count restarted to decrease rapidly to less than 20.000/μL despite adequate correction of romiplostim dosage, and a third‐line therapy was required. Nonetheless, other TPO‐RAs such as eltrombopag and avatrombopag were not exploitable because they are strictly metabolized by the liver enzyme cytochrome P450 3A4 isoform (CYP3A4), the same enzyme involved in the metabolism of bictegravir—emtricitabine—tenofovir alafenamide [16]. The most significant concern was the potential drug interaction leading to hepatic toxicity, impacting effectiveness of both essential therapies and increasing side effects. A safer and more efficient therapeutic approach was necessary.

**FIGURE 1:**
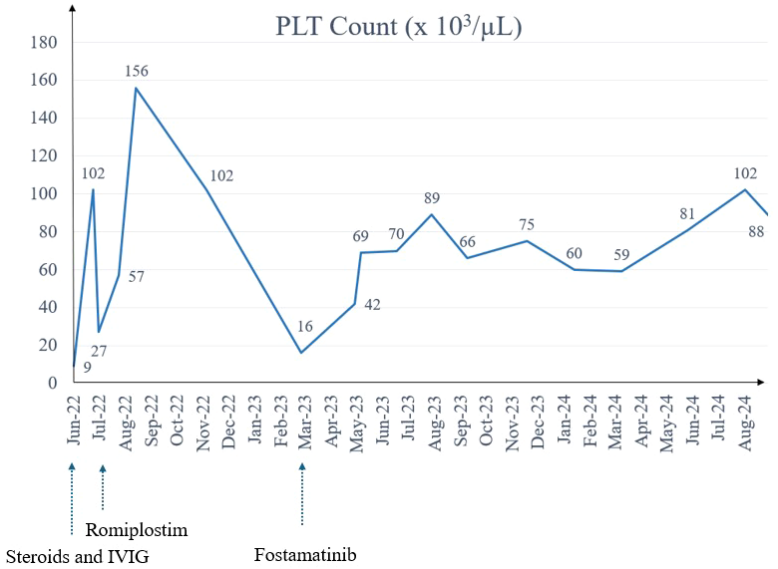
Timeline in months depicting clinical care of a 50 years‐old male HIV positive patient with relapsed and refractory Immune Thrombocytopenia (ITP). The Day 0 corresponds to patient hospitalization. The variation of platelets count is presented in relation to the clinical management. IVIG, intravenously immunoglobulin.

Therefore, given its safer interaction profile, in agreement with the infectious disease consultant, fostamatinib, a first‐in‐class Spleen Tyrosin Kinase Inhibitor (Syk‐I) indicated in chronic ITP, was initiated. Clinically, before fostamatinib administration, the patient presented with lower limbs petechiae and few oral cavity ecchymosis. Fostamatinib was administered initially at a first dosage of 100 mg orally twice daily and subsequently it was increased to 150 mg twice daily. This treatment regimen successfully induced a complete response in the patient with a rapid resolution of mucocutaneous haemorrhagic diathesis. This response has been sustained over a two‐year period. The kinetics of platelet count improvement demonstrated a progressive and consistent increase, with initial stabilization observed within the first few weeks of therapy. Over the subsequent months, platelet levels demonstrated a steady upward trend, stabilizing within a range that, while slightly below the normal threshold, represented a significant clinical improvement and maintained a level sufficient to prevent bleeding complications. This gradual but stable increase is illustrated in the accompanying Figure 1, which provides a visual representation of the timeline and magnitude of platelet normalization.

The efficacy of fostamatinib can be attributed to its unique mechanism of action as an inhibitor of Syk. By targeting Syk, fostamatinib disrupts the signaling pathways involved in antibody‐mediated platelet destruction, thereby reducing platelet clearance by macrophages in the spleen [15]. This targeted approach not only enhances platelet survival but also minimizes the risk of systemic immunosuppression, a common drawback of alternative therapies for ITP.

Importantly, the therapy was well‐tolerated, with no reported adverse effects such as elevated blood pressure, diarrhea, or hepatotoxicity throughout the treatment duration. While fostamatinib is generally associated with a favorable safety profile, potential adverse drug reactions include hypertension, gastrointestinal disturbances (such as diarrhea or nausea), and hepatic enzyme elevation [12]. Regular monitoring of blood pressure and liver function is recommended during treatment to detect and manage these potential side effects proactively. Interestingly, this drug does not affect kidneys, thus no dose adjustment is required in case of any grade of renal insufficiency. In this case, the absence of complications such as fatigue, gastrointestinal disturbances, or liver function abnormalities not only highlights the tolerability of fostamatinib, but also underscores its suitability as a long‐term treatment option for ITP. According to infectious disease follow‐up, during the patient clinical course, CD4 and CD3 T‐ lymphocyte counts were tested routinely resulting in normal range as well as HIV‐RNA was tested routinely and resulted negative even after fostamatinib treatment. Moreover, no ART‐related adverse drug reactions, such as fatigue, nausea, diarrhea and sleep disturbances, have been reported. This favorable safety profile significantly enhanced the patient's quality of life, enabling him to maintain normal daily activities without interruptions or discomfort commonly associated with adverse drug reactions.

## 4 Discussion and Conclusion

This rare case of a 50‐year‐old male HIV patient with severe immune thrombocytopenia highlights how challenging the ITP treatment can be due to ART interactions. The uniqueness of this case report stems from the innovative use and therapeutic potential of fostamatinib in relapsed and refractory ITP in the context of HIV infection. To the best of our knowledge, this is the first evidence of fostamatinib application in this setting during ART, and this case study demonstrates its effectiveness and its safe drug‐to‐drug interaction profile. In conclusion, our findings suggest that fostamatinib may represent an innovative, secure, and valid therapeutic option in HIV patients affected by relapsed and refractory ITP.

## Author Contributions

**Matteo Bellia:** conceptualization, data curation, methodology, writing – original draft. **Riccardo Moia:** methodology, supervision, validation, writing – review and editing. **Mattia Schipani:** methodology, visualization, writing – review and editing. **Carola Sella:** formal analysis, methodology, visualization, writing – review and editing. **Mariangela Greco:** supervision, visualization, writing – review and editing. **Wael Al Essa:** methodology, writing – review and editing. **Abdurraouf Mokhtar Mahmoud:** data curation, supervision, writing – review and editing. **Gianluca Gaidano:** conceptualization, project administration, supervision, validation, writing – review and editing. **Andrea Patriarca:** conceptualization, data curation, project administration, writing – review and editing.

## Ethics Statement

The authors have nothing to report.

## Consent

Written informed consent was obtained from the patient to publish this report in accordance with the journal patient consent policy.

## Conflicts of Interest

Dr. Riccardo Moia: speaker bureau and/or advisory board for Beigene, AbbVie, Astra Zeneca, Johnson & Johnson. Dr. Andrea Patriarca: speaker bureau and/or advisory board for Sanofi, Sobi, Pfizer, Incyte, Alexion, Takeda, Novartis, BMS. Prof. Dr. Gianluca Gaidano: speaker bureau and/or advisory board for AbbVie, Astra Zeneca, BeiGene, Hikma, Johnson and Johnson, and Lilly. The other authors declare no conflicts of interest.

## Data Availability

The data that support the findings of this study are available on request from the corresponding author. The data are not publicly available due to privacy or ethical restrictions.
